# Prediction of Hormone-Binding Proteins Based on K-mer Feature Representation and Naive Bayes

**DOI:** 10.3389/fgene.2021.797641

**Published:** 2021-11-23

**Authors:** Yuxin Guo, Liping Hou, Wen Zhu, Peng Wang

**Affiliations:** ^1^ Key Laboratory of Computational Science and Application of Hainan Province, Haikou, China; ^2^ Yangtze Delta Region Institute, University of Electronic Science and Technology of China, Quzhou, China; ^3^ Key Laboratory of Data Science and Intelligence Education, Hainan Normal University, Ministry of Education, Haikou, China; ^4^ School of Mathematics and Statistics, Hainan Normal University, Haikou, China; ^5^ Beidahuang Industry Group General Hospital, Harbin, China

**Keywords:** hormone binding protein, feature selection, protein classification, k-mer, naive Bayes model

## Abstract

Hormone binding protein (HBP) is a soluble carrier protein that interacts selectively with different types of hormones and has various effects on the body’s life activities. HBPs play an important role in the growth process of organisms, but their specific role is still unclear. Therefore, correctly identifying HBPs is the first step towards understanding and studying their biological function. However, due to their high cost and long experimental period, it is difficult for traditional biochemical experiments to correctly identify HBPs from an increasing number of proteins, so the real characterization of HBPs has become a challenging task for researchers. To measure the effectiveness of HBPs, an accurate and reliable prediction model for their identification is desirable. In this paper, we construct the prediction model HBP_NB. First, HBPs data were collected from the UniProt database, and a dataset was established. Then, based on the established high-quality dataset, the k-mer (K = 3) feature representation method was used to extract features. Second, the feature selection algorithm was used to reduce the dimensionality of the extracted features and select the appropriate optimal feature set. Finally, the selected features are input into Naive Bayes to construct the prediction model, and the model is evaluated by using 10-fold cross-validation. The final results were 95.45% accuracy, 94.17% sensitivity and 96.73% specificity. These results indicate that our model is feasible and effective.

## Introduction

With the rapid development of society, people have higher and higher requirements for medical and health care (Lin, 2020). Therefore, it is urgent to learn more about the structure and function of proteins in order to explain more of the meaning of life and promote the development of biomedicine and other fields (Wang et al., 2020a; Qu et al., 2021). However, there is a difficulty in the current research, that is, how to use its sequence information to predict proteins effectively. Although effective prediction of protein sequences can be made using physical, chemical and biological experiments, these methods are costly and time consuming.

Hormone binding proteins (HBPs) are carrier proteins that bind specifically to targeted hormones and were first identified in the plasma of pregnant mice, rabbits and humans (Mortezaeefar et al., 2019; Niu et al., 2021a). They are involved in hormonal regulation in living organisms. HBPs not only regulate the amount of hormones reaching the target cell to produce the desired effect (Wang et al., 2018) but also regulate non-protein-binding or free-circulating active steroid hormones, which are thought to be the main gatekeepers of steroid effects. Sexual HBPs, mainly produced in the liver, combine with sexual steroid hormones to regulate their bioavailability. The incorrect expression of HBPs, however, can cause various diseases (Tan et al., 2019).

Therefore, understanding the function and regulatory mechanism of HBPs has become very important. Accurately identifying HBPs is the first step in studying their function. Traditional HBPs identification methods involve wet biochemical experiments, such as immunoprecipitation, chromatography, or cross-linking (Sohm et al., 1998; Zhang and Marchant, 1999; Einarsdóttir et al., 2014; Cheng et al., 2016; Fang et al., 2019). These experimental methods are time-consuming and expensive, and with the discovery of a large number of protein sequences, it is difficult to determine HBPs through biochemical experiments. Therefore, it is necessary to establish an effective recognition model to identify HBPs (Akbar et al., 2020). The description of the characteristics of the protein sequence method contains a lot of information, such as the chemical and physical properties of amino acids, sequence characteristics, feature extraction algorithm for classification algorithm which has great impact on the design and the classification of results. Generally, prediction techniques based on machine learning consist of three steps: feature extraction, construction of predictors, and performance evaluation (Liu, 2017; Wang et al., 2018; Zhang et al., 2019). In 2018, Tang et al. (Hua et al., 2018). developed a method based on support vector machines to identify HBPs, which uses the optimal characteristic coding protein obtained by using the optimized dipeptide composition. Subsequently, Basith et al. developed the computational predictor iGHBP, which combined the dipeptide composition and the value of the amino acid index to obtain the optimal selection and predict the construction model (Basith et al., 2018). In this paper, we constructed a prediction model, HBP_NB, to correctly identify HBPs. First, the k-mer (Liu et al., 2008; Christopher et al., 2013; Liu et al., 2015a; Manavalan et al., 2019) method was used to obtain the frequency characteristics of protein sequences, and then the F-score value method was used to select the feature subset. Finally, input the obtained features into Naive Bayes (Gong and Tian, 2010; He et al., 2010; Gumus et al., 2014; Hu et al., 2020; Hu et al., 2021a; Hu et al., 2021b) to construct the prediction model.

## Materials and Methods

### Main Process of the Article

Machine learning frameworks have been used to identify multiple protein types, such as DNA binding proteins (Zeng et al., 2015; Qu et al., 2017; Shen and Zou, 2020), RNA binding proteins (Xiao et al., 2017; Lei et al., 2021), lncRNA interacting proteins (Zhang et al., 2017; Liu, 2020), and drug targets (Yan et al., 2016; Wang et al., 2020b; Wang et al., 2020c). Since the recognition of protein sequences includes two important steps sequence feature extraction and classifier selection the effective combination of feature extraction algorithms and classifiers has also been extensively studied (Zhang et al., 2016). In this paper, we propose a predictive model for identifying hormone-binding proteins based on Naïve Bayes.

**FIGURE 1:**
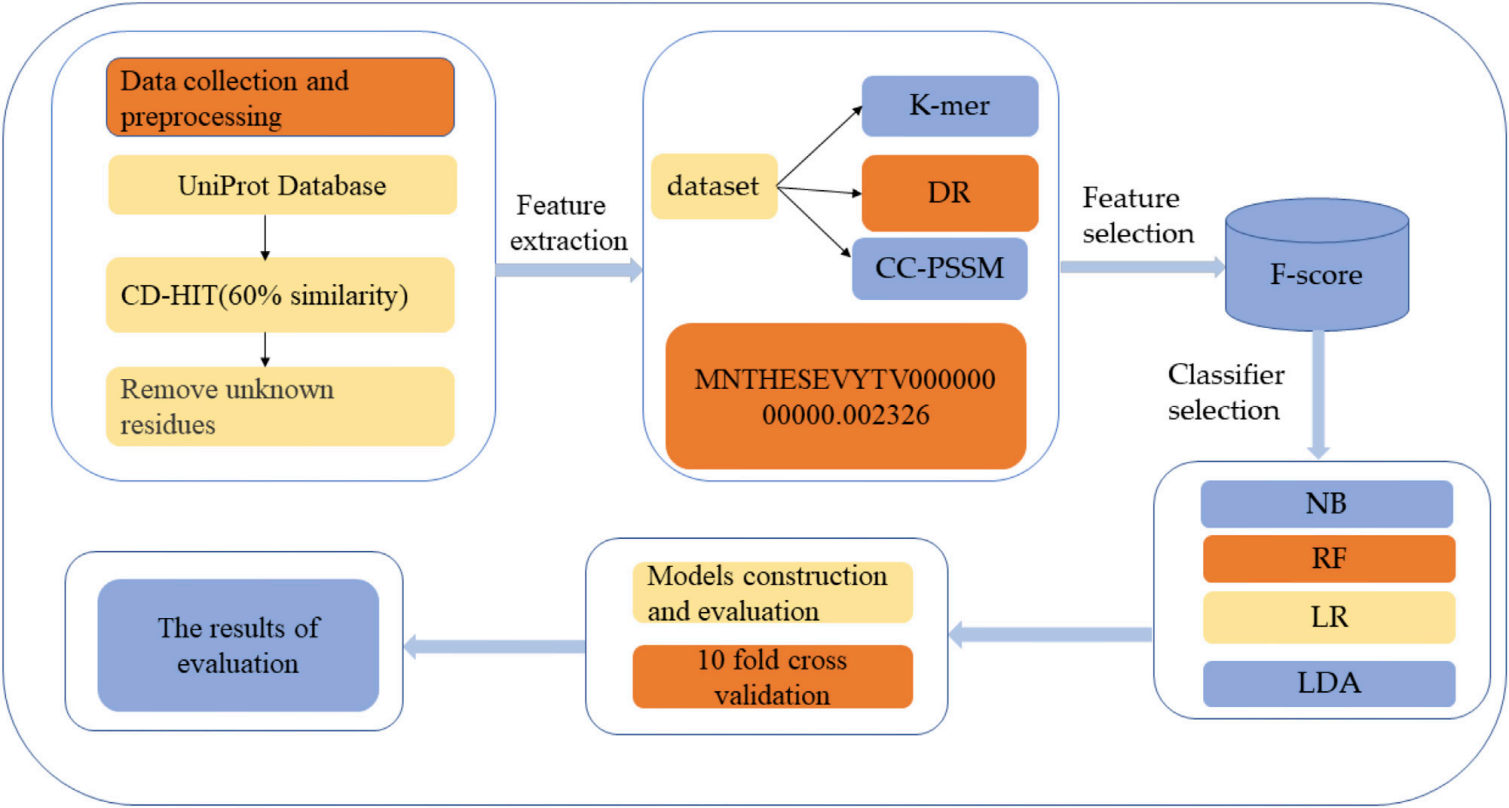
Structure flow chart. The first step is to search and download HBPs and non-HBPs from the protein resource database and then use CD-HIT to perform protein de-redundancy operations. The threshold is set to 60%. Finally, protein sequences containing unknown residues are removed to generate the final protein dataset. The second step is to extract features of the protein, and the third step is to use different classification methods to classify the selected features.

HBPs prediction analysis was carried out through the following five steps: 1) HBPs and non-HBPs were searched and downloaded from UniProt, and the similarity threshold of protein sequences was set by the CD-HIT program to construct a high-quality dataset (Zou et al., 2020); 2) feature extraction of protein sequences was performed using the k-mer feature coding method; 3) the extracted features were selected to improve the accuracy of classification; 4) different classification methods were used to classify and predict the selected feature subset and select the best classification methods; and 5) Performance evaluation. Figure 1 shows the structural framework for identifying HBPs in this paper. This section will introduce dataset establishment, feature selection methods and classification methods in detail.

### Dataset

It is necessary to collect sufficient correlation function data as the basis of statistical model prediction. Therefore, it is first necessary to construct an objective dataset to ensure the effectiveness and robustness of the model. Therefore, we adopt the benchmark dataset constructed by Tang et al. (Tang et al., 2018). To build this dataset, follow these steps. The first step was to search and collect HBPs from UniProt (Bairoch et al., 2009; Schneider, 2012) and to generate the original HBPs dataset by selecting the hormone binding keywords in the molecular function items of the gene body (Ashburner et al., 2000). Consequently, 357 HBPs with manual annotation and review were selected. In the second step, to avoid the high similarity of protein sequences affecting the results, we used the CD-HIT (Li and Godzik, 2006; Fu et al., 2012) program to set the truncation threshold to 0.6 to remove highly similar HBPs sequences. In the third step, when the protein sequence in the dataset contains unknown residues (such as “X,” “Z,” and “B”), it will affect the model prediction results, so protein sequences containing unknown residues need to be excluded. After the above steps, a total of 122 HBPs were obtained, which were regarded as positive data. As a control, 121 non-HBPs were randomly selected from UniProt as negative data using a similar selection strategy. The data of the model can be freely download from https://github.com/GUOYUXINXIN/-. The benchmark dataset can be expressed as:

$$D=D_{p}\cup D_{n}$$ (1)

Among them, subset $D_{p}$ contains 122 HBPs, and subset $D_{n}$ contains 121 non-HBPs.

### Feature Extraction

Protein sequence is a string generated by the permutation and combination of 20 English letters with different lengths. Currently, general machine learning algorithms can only deal with feature vectors, so when machine learning methods are used, protein sequences need to be transformed into numerical vectors representing the characteristics of protein sequences. As the first step in building a biological sequence analysis model, feature extraction is an important part of correctly predicting protein sequences, an efficient feature extraction method can obtain a high performance classification model. The extracted features should not only retain the protein sequence information to the maximum extent, but also have a greater correlation with protein classification. Given a protein sequence, express it as:

$$P=R_{1}R_{2}R_{3}\cdots R_{L}$$ (2)

where P stands for protein sequence, $R_{i}$ represents the ith amino acid residue of protein P(i=1,2,⋯,L) .

#### K-Mer

K-mer (Liu et al., 2015b; Niu et al., 2021b) is the most basic method of expressing protein sequences as digital vectors (Liu et al., 2016), in which k-mer frequency coding refers to the occurrence frequency of all possible nucleotide sequences with k length in a given sequence (Liu et al., 2015c; Bin et al., 2017). The k-mer feature extraction algorithm is used to convert the protein sequence into a vector with a fixed length, which is used as the input vector of the machine learning classifier. For example, setting k to 2 produces a 400-dimensional vector (AA,AC,AD,⋯,AY,YA,YC,⋯,YY) . To avoid the problem of overfitting, we generally set k<4 because when k>4 , more dimensions will be generated, resulting in dimension disaster (Wei et al., 2019). Therefore, we set k to 3 so that the input protein sequence could be converted into a vector with 8,000 dimensions of fixed length.

#### Distance-Based Residual

DR (Liu et al., 2014) is a feature expression method based on protein sequences that uses the distance between residue pairs to represent the feature vector of the protein. The feature vector is expressed by calculating the number of occurrences of residual pairs within a certain distance threshold. The feature vector dimension obtained by the DR feature extraction method is $20+20\times 20\times d_{MAX}$ dimensions, where in 20 in $20+20\times 20\times d_{MAX}$ represents the types of amino acids that make up the protein; $d_{MAX}$ is a distance threshold that can be set manually, which represents the maximum distance between pairs of amino acid residues.

#### Profile-Based Cross-Covariance

Since machine learning-based technologies such as random forest (RF) and logistic regression (LR) require the input of fixed-length vectors as input vectors for training, it is necessary to convert protein sequences of different lengths into fixed length vectors as input vector machine learning. Because each residue in a protein has many physical and chemical properties, protein sequences can be regarded as time series with similar properties. Therefore, CC-PSSM (Dong et al., 2009) is used in this article to convert protein sequences of different lengths into fixed length vectors. PSSM algorithm is a common algorithm in the field of bioinformatics, known as the “position-specific scoring matrix,” which can store the evolutionary information of protein sequences so that it can be used for protein prediction. It is a matrix that calculates the percentage of different residues at each position in a multi sequence alignment, the matrix size is L×20 ( L for protein sequence length). Among them, CC is a measure of correlation between two different properties of amino acid residues and can be calculated using the following equation:

$$CC\left(i1,i2,lag\right)=\sum_{j=1}^{L-lag}\left(S_{i1,j}-{\bar{S}}_{i1}\right)\left(S_{i2,j+lag}-{\bar{S}}_{i2}\right)/\left(L-lag\right)$$ (3)

where i1,i2 represents amino acids, and ${\bar{S}}_{i1},{\bar{S}}_{i2}$ represents the average score of i1,i2 along the protein sequence. LAG is the maximum lag, lag is an integer value from 1 to LAG , and the total number of CC variables is 380×LAG . In this paper, we set the value of LAG to 2 to obtain a 720(380×2) -dimensional vector.

### Feature Selection

When the feature size is large, there may be irrelevant features or inter-dependence between features, which will easily affect the accuracy of the prediction results. In particular, the more feature dimensions, the more likely it is to lead to “dimension disaster,” model complexity and model generalization ability decline. Therefore, removing irrelevant or redundant features through feature selection can improve the accuracy of classification performance and reduce the running time of the model (Polat and Güneş, 2009; Quan et al., 2016; Zou et al., 2016; Guohua and Jincheng, 2018; Wei et al., 2018; Riaz and Li, 2019; He et al., 2020). In this paper, the F-score value is used to select the optimal feature (Chen and Lin, 2008; Cheng et al., 2019; Wei et al., 2019), which is a method to measure the distinguishing ability of features between the two categories, and the most effective feature selection can be achieved through this method. Therefore, we can use (Eq. 4) to describe the contribution of each feature and perform feature selection:

$$F\left(i\right)=\frac{s_{b}^{2}\left(i\right)}{s_{w}^{2}\left(i\right)}$$ (4)

where F(i) is the score of the ith feature of the F-score. Generally, the larger the value of F(i) is, the stronger the ability to recognize samples. $s_{w}^{2}\left(i\right)$ is the intragroup variance, and $s_{b}^{2}\left(i\right)$ is the intergroup variance. Their calculation formula is as follows:

$$\left\{ \begin{array}{l} s_{b}^{2}\left(i\right)=\frac{ss_{b}\left(i\right)}{K-1}\\ s_{w}^{2}\left(i\right)=\frac{ss_{w}\left(i\right)}{N-K} \end{array}\right.$$ (5)

where $ss_{b}\left(i\right)$ is the sum of squares between groups; $ss_{w}\left(i\right)$ is the sum of squares within the group; K is the total number of classes; and N is the total number of samples.

### Classifier

In this paper, Naive Bayes, Random forests, logistic regression, linear discriminant and other classification algorithms are used to predict HBPs.

#### Naïve Bayes

The Naive Bayes method is a classification method based on Bayes’ theorem and the assumption of the independence of characteristic conditions. It is characterized by combining prior probability and posterior probability and a very widely used algorithm. The main idea of the naive Bayes classifier is to solve the posterior probability P(Y|X) through joint probability modeling and use Bayes’ theorem. Then, the category corresponding to the largest posterior probability is used as the predicted category. Suppose there is a sample dataset $D=\{d_{1},d_{2},\cdots ,d_{n}\}$ , the feature dataset corresponding to the sample dataset is $X=\{x_{1},x_{2},\cdots ,x_{d}\}$ , features are independent and random, and the class variable is $Y=\{y_{1},y_{2},\cdots y_{m}\}$ . According to the Naive Bayes algorithm, the posterior probability of the sample belonging to category y can be expressed as:

$$P\left(Y|X\right)=\frac{P\left(Y\right)P\left(X|Y\right)}{P\left(X\right)}$$ (6)

Where P(Y) is the prior probability, Naive Bayes is based on the independence of each feature. In the case of a given category, Eq. 6 can be further expressed as the following equation:

$$P\left(X|Y=y\right)=\prod_{i=1}^{d}P\left(x_{i}|Y=y\right)$$ (7)

The posterior probability can be calculated from the above two Eqs 6, 7:

$$P\left(Y|X\right)=\frac{P\left(Y\right)\prod_{i=1}^{d}P\left(x_{i}|Y\right)}{P\left(X\right)}$$ (8)

Since the magnitude of P(X) is fixed, when comparing the posterior probability, only the molecular part of the above equation can be compared. Therefore, a naive Bayesian calculation of sample data belonging to category $y_{i}$ can be obtained:

$$P\left(y_{i}|x_{1},x_{2},\cdots ,x_{d}\right)=\frac{P\left(y_{i}\right)\prod_{j=1}^{d}P\left(x_{j}|y_{i}\right)}{\prod_{j=1}^{d}P\left(x_{j}\right)}$$ (9)

#### Random Forests

RF is a flexible, easy-to-use machine learning algorithm that contains multiple decision trees. It is an optimized version of bagging (Su et al., 2019; Zeng et al., 2020). The idea of bagging is to vote on the results of multiple weak classifiers to combine them into a strong classifier, thereby improving the prediction accuracy of the model. In the training phase, RF uses the bootstrap sampling method to collect multiple different subsets from the input training dataset and then uses the different collected subsets to train the internal decision tree. Then, in the prediction phase, RF votes for the prediction results of multiple internal decision trees and then outputs the prediction results. Its advantages are as follows: 1) it can process high-dimensional data without feature selection; 2) accuracy can be maintained even if many of the features are missing; and 3) it has a fast training speed (Jiao et al., 2021).

#### Logistic Regression

As a classification model, LR can deal with the 0/1 classification problem because of the nonlinear factor introduced by the sigmoid function. The image of the logical function is an S-shaped curve with values between (0, 1). The farther away from 0 a function is, the closer to 0 or 1 the value of the function will be. Therefore, this feature can be used to solve the problem of binary classification. The function formula is as follows:

$$g\left(z\right)=\frac{1}{1+e^{-z}}$$ (10)

Among them, $z=\theta^{T}x=\sum_{i=0}^{n}\theta_{i}x_{i}=\theta_{0}x_{0}+\theta_{1}x_{1}+\theta_{2}x_{2}+\cdots +\theta_{n}x_{n}$ ; therefore, the predictive function of logistic regression can be expressed as:

$$h_{\theta }\left(x\right)=g\left(\theta^{T}x\right)=\frac{1}{1+e^{-\theta^{T}x}}$$ (11)

#### Linear Discriminant Analysis

LDA is a classical linear learning method, also known as “Fisher” discriminant analysis in dichotomies. Unlike the perception machine, the principle of LDA is dimension reduction. In other words, given a set of training samples, the article tries to sample projections to a straight line, keeping the points with the same classification as close as possible and the classification of different points as far apart as possible, i.e., maximizing and minimizing the variance between variance. LDA can, therefore, make use of sample points in the projection line (or projection location) to determine the type of sample.

### Performance Evaluation

In this article, we use the specificity (SP), sensitivity (SN), accuracy (ACC) (Yang et al., 2021) and Matthews correlation coefficient (MCC) to evaluate our proposed method (Snow et al., 2005; Cheng et al., 2018), which can be expressed as:

1. Accuracy: ACC represents the probability that all positive and negative samples will be correctly predicted.

$$ACC=\frac{TP+TN}{TP+TN+FN+FP}$$ (12)

2. Sensitivity: SN represents the probability that the actual hormone-binding protein is predicted to be a hormone-binding protein.

$$SN=\frac{TP}{TP+FN}$$ (13)

3. Specificity: SP represents the probability that a non-hormone-binding protein is predicted to be a non-hormone-binding protein.

$$SP=\frac{TN}{TN+FP}$$ (14)

4. MCC: MCC represents the reliability of the algorithm results.

$$MCC=\frac{TP\times TN-FP\times FN}{\sqrt{\left(TP+FP\right)\left(TP+FN\right)\left(TN+FP\right)\left(TN+FN\right)}}$$ (15)

5 Precision: Indicates how many of the samples predicted to be positive are true positive samples.

$$pre=\frac{TP}{TP+FP}$$ (16)

6. F1-Score: The F1 score is balanced by taking into account both accuracy and recall, so that both are maximized at the same time.

$$F1-Socre=\frac{2\times pre\times recell}{pre+recell}$$ (17)

Where, the recall rate is: $recell=\frac{TP}{TP+FN}$

7. The ROC curve: Receiver operating characteristic curve (the area under the curve is AUROC), *X*-axis is false positive rate (FPR), *Y*-axis is true positive rate (TPR):

$$TPR=\frac{TP}{TP+FN}$$ (18)

$$FPR=\frac{FP}{FP+TN}$$ (19)

8. PRC: PRC takes precision rate as *Y*-axis and recall rate as *X*-axis.

Where TP refers to the model correctly predicting positive category samples; FP refers to the model incorrectly predicting negative category samples as positive category; TN refers to the model correctly predicting negative category samples; and FN refers to the model incorrectly predicting positive category samples as negative category (Ding et al., 2020a; Ding et al., 2020b).

**FIGURE 2:**
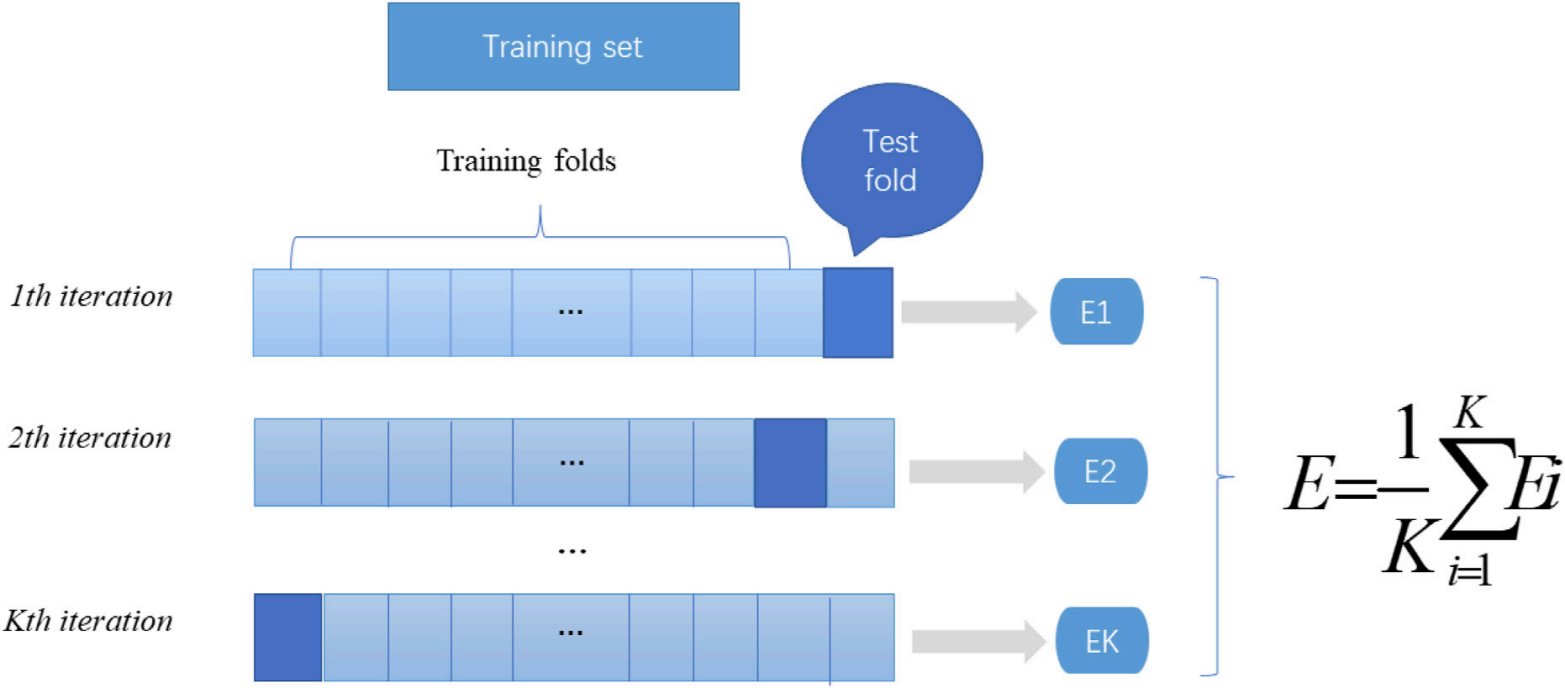
K-fold cross-validation diagram. Divide the data into K parts, where k-1 parts are used as the training dataset, and the remaining part is used as the test set. The mean value of the results of the k groups is calculated as the performance index of the current k-fold cross-validation evaluation model.

In machine learning, a test set is needed to test the model and describe its generalization ability. However, in practical applications, due to the limited number of datasets, cross validation is used as a test method. There are three types of cross validation: K-fold cross validation, fold cross validation and independent data verification. In this article, we use K-fold cross-validation to test the constructed model. K-fold cross-validation divides the training data into K parts, of which (K-1) pieces of data are used to train the model, and the remaining 1 piece of data is used to evaluate the quality of the model. This process is cycled K times, and the K evaluation results obtained are combined, such as averaging or voting. The flow chart of K-fold cross verification is shown in Figure 2.

## Results and Discussion

In machine learning, the predicted results of the model can be tested through cross-validation. In this article, we use 10-fold cross-validation to evaluate the built model.

### Performance Comparison of Different Feature Expression Methods

According to the feature extraction part, protein sequences are transformed into feature vectors of different sizes through different feature extraction methods. Therefore, in this study we tested the performance of three feature extraction methods: k-mer (K = 2), k-mer (K = 3), DR and CC-PSSM.

First, use the F-score feature selection method to reduce the dimensionality of the feature vectors obtained by different feature extraction methods to 250 dimensions, then use the selected best feature vector as the input vector of the naive Bayes algorithm and perform 10-fold cross-validation, and finally draw forecast results. The prediction results are shown in Table 1 (the maximum value is in bold). As shown in Table 1, the k-mer (k = 3) feature extraction algorithm used in this model performs best in all indicators, among which the values of ACC, MCC, SP and SN are, respectively, 95.45,91.36, 96.73, and 94.17%. These results prove the validity of our model.

**TABLE 1:** Prediction results of different feature extraction algorithms based on the Bayesian classifier.

Feature extraction	SN(%)	SP(%)	ACC(%)	MCC(%)	AUROC(%)	PRC(%)
K-mer(k = 3)	**94.17**	**96.73**	**95.45**	**91.36**	**95.17**	**96.55**
K-mer(k = 2)	65.51	78.46	71.96	44.50	77.89	76.97
DR	83.46	37.12	60.39	25.64	66.35	75.99
CC-PSSM	64.10	80.13	72.09	45.29	78.24	80.27

### Comparison With Other Classifiers 

**FIGURE 3:**
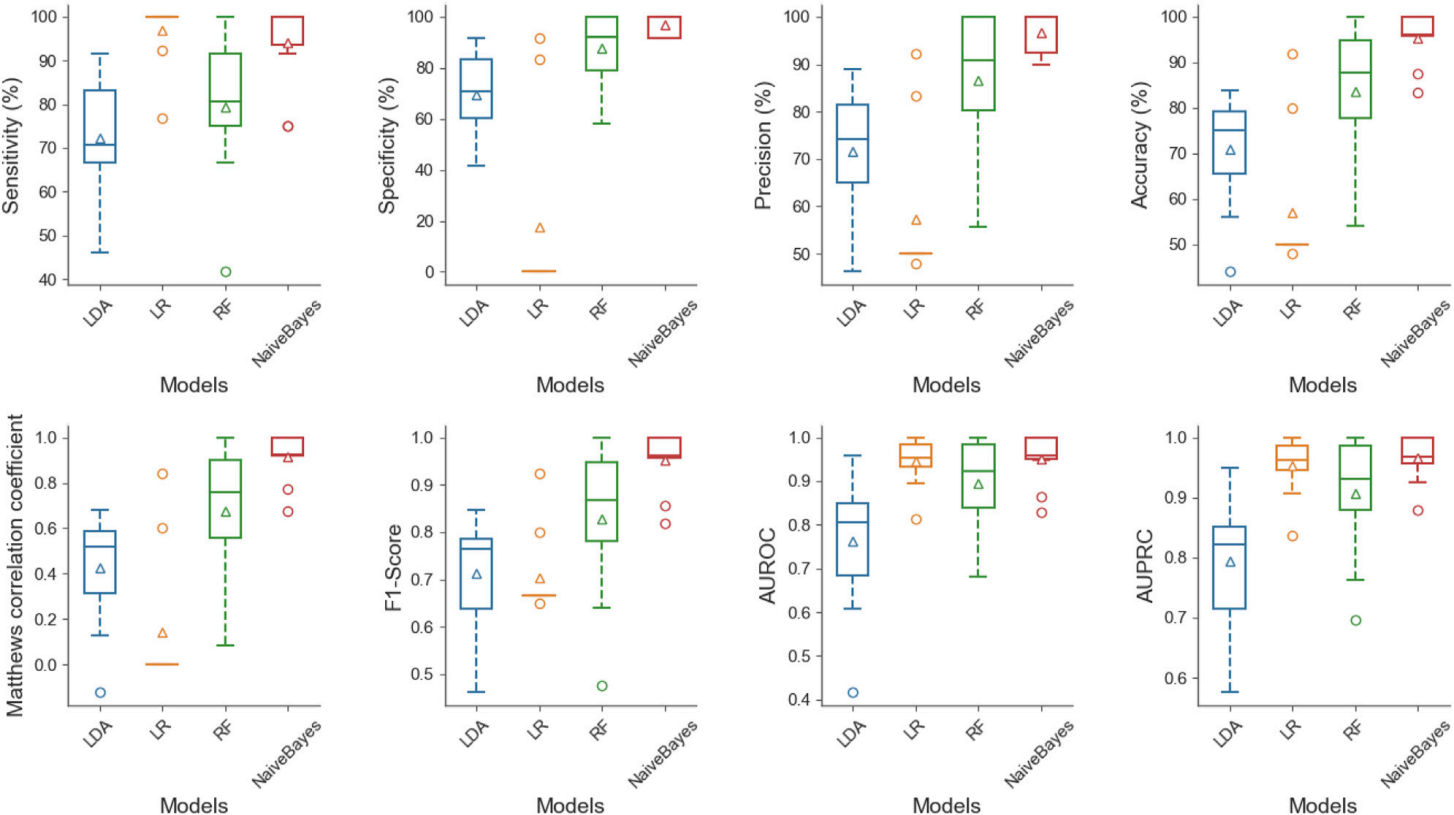
Boxplot diagram of different classification models, this figure shows the distribution of LDA, LR, RF and NB under SN, SP, accuracy, ACC, MCC, F1-Score, AUROC and AUPRC successively from left to right and from top to bottom. At the same time, it can be seen from the figure that NB can achieve good results under different indicators.

**FIGURE 4:**
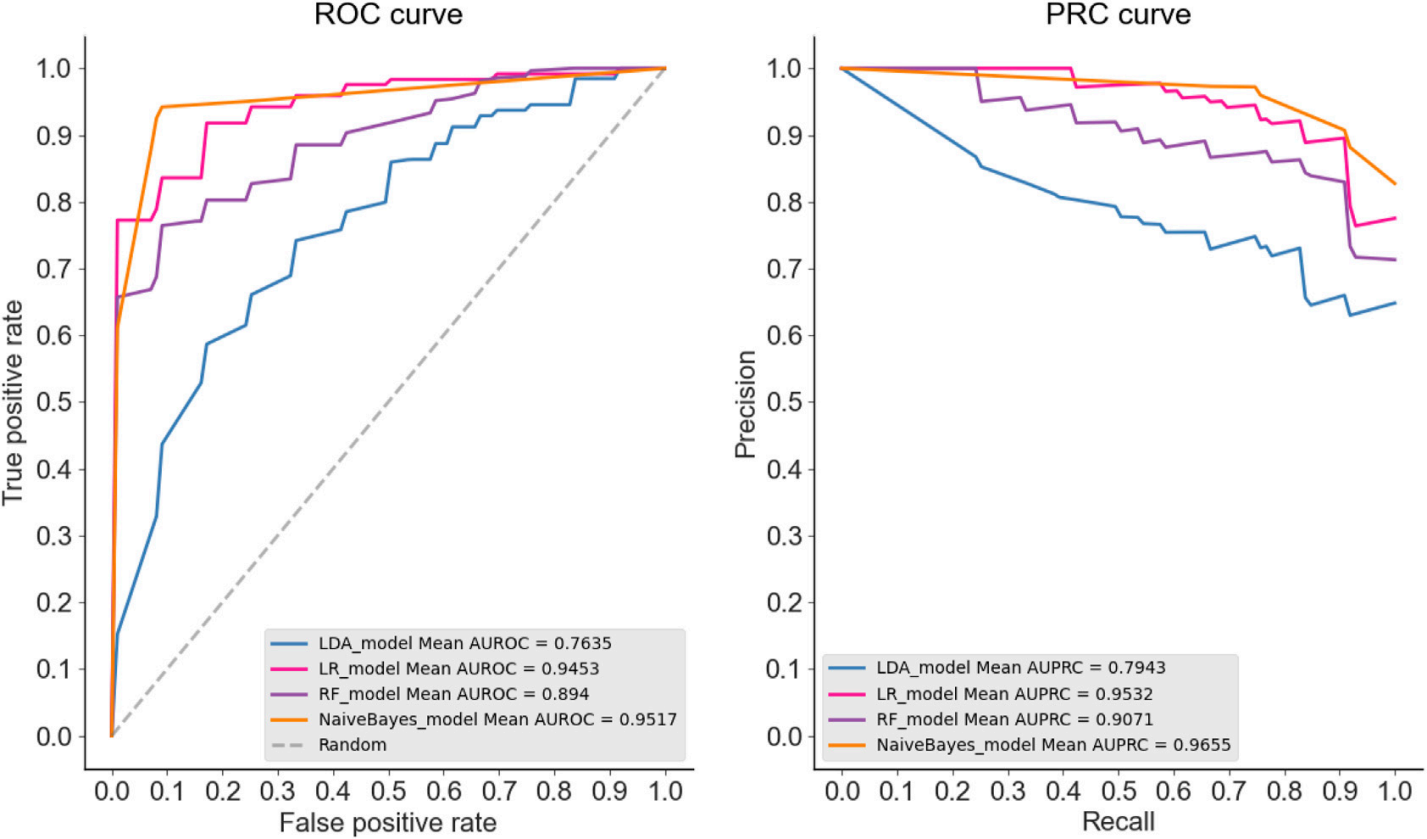
As can be seen from the ROC curves and PRC curves of different classification models, the ROC curves of LDA, RF, LR and NB are 0.7635, 0.894 and 0.9453, respectively. The dotted line represents the ROC curve of a completely random classifier, and the ROC curve of a good classifier should be as far away from the dotted line as possible, as close as possible to the upper left corner; The PRC curve values of LDA, RF, LR and NB were 0.7943, 0.9071, 0.9532 and 0.9655, respectively, the closer the curve was to the upper right corner, the better the model classification ability was. Therefore, we constructed the final model based on NB.

To show the superiority of naive Bayes in HBPs recognition, we can compare the HBPs recognition performance of different classification algorithms based on the same feature subset (i.e. 250 optimal features). In this paper, we used the constructed HBP_NB model to perform performance comparison with RF, LDA, Logistic regression and other models under the condition of 10-fold cross-validation, and the comparison results are shown as follows. Table 2 shows the specific values of different classification models under SN, SP, ACC, MCC and other indicators (the maximum values are in bold). As can be seen from Table 2, HBP_NB prediction model achieved better results than other classification algorithms in identifying hormone-binding proteins, in which ACC, MCC, SN and SP were 95.45, 91.36, 94.17 and 96.73%, respectively. Figures 3, 4 respectively show the boxplot diagram of different models, ROC and PRC curves schematic diagram. These results show that our model has good classification ability. Therefore, we construct the final model based on naive Bayes. Where, the line in the middle of the box in the boxplot is the median of the data, representing the average level of the sample data; The top of the box represents the upper quartile and the bottom quartile represents the lower quartile, which means the box contains 50% of the data, so the width of the box reflects, to some extent, how much the data fluctuates; at the same time, the lines above and below the box represent the maximum and minimum values of data. The ROC curve is a curve that evaluates the effect of binary model on positive category prediction. *X*-axis is false positive rate (FPR), *Y*-axis is true positive rate (TPR), which indicates that the optimal classifier with the best performance is located in the upper left corner of the image (coordinate 0,1), and the area under its ROC curve is AUROC, with an area value between 0,1. PRC takes presion rate as *Y*-axis and recall rate as *X*-axis, and lines are drawn according to changes in the value of probability threshold. The ideal model would be at the point (1,1). The model with excellent performance is as close to this point as possible.

**TABLE 2:** Performance comparison of different classifiers under 10-fold cross validation

Classifier	SN(%)	SP(%)	ACC(%)	MCC(%)	AUROC(%)	PRC(%)
NB	94.17	**96.73**	**95.45**	**91.36**	**95.17**	**96.55**
RF	77.95	87.57	82.71	66.26	89.45	91.19
LDA	72.24	70.13	71.20	43.08	94.53	95.32
LR	**96.92**	17.50	57.00	14.42	76.35	79.43

### Performance Comparison With the Existing Optimal Algorithm

This section compares the model constructed in the article with other existing methods, in which the results of HBPred (Hua et al., 2018) and iGHBP (Basith et al., 2018) are directly obtained from the literature. The comparison results are shown in Table 3 (the maximum value is in bold). As seen from Table 3, the HBP_NB model constructed in this paper has the best performance in all indicators, among which ACC, SP and SN have reached maximum values of 95.45, 96.73 and 94.17%, respectively. The effect is significantly better than that of the other two methods, which also proves the effectiveness of the HBP_NB model constructed in this paper.

**TABLE 3:** Comparison of our method with other published methods.

Methods	SN(%)	SP(%)	ACC(%)
HBPred Hua et al. (2018)	80.43	56.52	68.48
iGHBP Basith et al. (2018)	86.96	47.83	67.39
HBP_NB	**94.17**	**96.73**	**95.45**

## Conclusion

As a carrier protein related to the regulation of hormones in the circulatory system, HBPs can cause various diseases when they are abnormally expressed. Therefore, it is very important to understand their function and regulatory mechanism, and the correct identification of HBPs is the first step in understanding their biological process and is necessary to further study their function. There is growing evidence that it is crucial to develop an efficient computational model to identify hormone-binding proteins. In this study, we used a reliable predictive model for HBP_NB to identify HBPs. First, the model uses the k-mer feature extraction method to extract the features of HBPs. Then, to remove redundancy and noise and improve the accuracy of model prediction, the F-score value is used to sort the features and select the optimal features. Secondly, the reduced feature set is input into naive Bayes classifier and the 10-fold cross validation is used to judge the quality of the prediction model. Finally, the accuracy, sensitivity and specificity of the HBP_NB model reached 95.45, 94.17 and 96.73%, respectively, in 10-fold cross validation. The feasibility and validity of our model are illustrated.

However, there is room for improvement in our current approach. Since the data set selected in this experiment is small, we will collect more data for model training and independent test set experiments in the future to improve the model’s robustness and generalization ability. At the same time, we will further learn more effective feature representation methods and classification algorithms to gain an in-depth understanding of machine learning and establish a more stable model. In addition, we also hope that our work can help scholars to study hormone binding proteins, to promote research on hormone-binding protein drugs.

## Data Availability

The original contributions presented in the study are included in the article/Supplementary Material, further inquiries can be directed to the corresponding authors.

## References

[B1] Akbar S. Khan S. Ali F. Hayat M. Qasim M. Gul S. (2020). iHBP-DeepPSSM: Identifying Hormone Binding Proteins Using PsePSSM Based Evolutionary Features and Deep Learning Approach. Chemometrics Intell. Lab. Syst. 204, 104103. 10.1016/j.chemolab.2020.104103

[B2] Ashburner M. Ball C. A. Blake J. A. Botstein D. Butler H. Cherry J. M. (2000). Gene Ontology: Tool for the Unification of Biology. Nat. Genet. 25 (1), 25–29. 10.1038/75556 10802651 PMC3037419

[B3] Bairoch A. Bougueleret L. Altairac S. Amendolia V. Zhang J. (2009). The Universal Protein Resource (UniProt) 2009. Nucleic Acids Res. 37 (Suppl. 1), D169–D174. 10.1093/nar/gkn664 18836194 PMC2686606

[B4] Basith S. Manavalan B. Shin T. H. Lee G. (2018). iGHBP: Computational Identification of Growth Hormone Binding Proteins from Sequences Using Extremely Randomised Tree. Comput. Struct. Biotechnol. J. 16, 412–420. 10.1016/j.csbj.2018.10.007 30425802 PMC6222285

[B5] Bin L. Hao W. Kuo-Chen C. (2017). Pse-in-One 2.0: An Improved Package of Web Servers for Generating Various Modes of Pseudo Components of DNA, RNA, and Protein Sequences. Nat. Sci. 9 (4), 67–91. 10.4236/ns.2017.94007

[B6] Chen Y. W. Lin C. J. (2008). Combining SVMs with Various Feature Selection Strategies Feature Extraction. Taipei, Taiwan: Studies in Fuzziness and Soft Computing.

[B7] Cheng L. Hu Y. Sun J. Zhou M. Jiang Q. (2018). DincRNA: a Comprehensive Web-Based Bioinformatics Toolkit for Exploring Disease Associations and ncRNA Function. Bioinformatics 34 (11), 1953–1956. 10.1093/bioinformatics/bty002 29365045

[B8] Cheng L. Shi H. Wang Z. Hu Y. Yang H. Zhou C. (2016). IntNetLncSim: an Integrative Network Analysis Method to Infer Human lncRNA Functional Similarity. Oncotarget 7 (30), 47864–47874. 10.18632/oncotarget.10012 27323856 PMC5216984

[B9] Cheng L. Yang H. Zhao H. Pei X. Shi H. Sun J. (2019). MetSigDis: a Manually Curated Resource for the Metabolic Signatures of Diseases. Brief Bioinform 20 (1), 203–209. 10.1093/bib/bbx103 28968812

[B10] Christopher F. B. Dongwon L. Mccallion A. S. Beer M. A. (2013). Kmer-SVM: a Web Server for Identifying Predictive Regulatory Sequence Features in Genomic Data Sets. Nucleic Acids Res. W1, W544–W556. 10.1093/nar/gkt519 PMC3692045 23771147

[B11] Ding Y. Tang J. Guo F. (2020). Identification of Drug-Target Interactions via Dual Laplacian Regularized Least Squares with Multiple Kernel Fusion. Knowledge-Based Syst. 204, 106254. 10.1016/j.knosys.2020.106254

[B12] Ding Y. Tang J. Guo F. (2020). Identification of Drug–Target Interactions via Fuzzy Bipartite Local Model. Neural Comput. Appl. 32 (D1), 1–17. 10.1007/s00521-019-04569-z

[B13] Dong Q. Zhou S. Guan J. (2009). A New Taxonomy-Based Protein Fold Recognition Approach Based on Autocross-Covariance Transformation. Bioinformatics 25 (20), 2655–2662. 10.1093/bioinformatics/btp500 19706744

[B26] Einarsdóttir I. E. Gong N. Jnsson E. Sundh H. Hasselberg-Frank L. Nilsen T. O. (2014). Plasma Growth Hormone-Binding Protein Levels in Atlantic salmonSalmo Salarduring Smoltification and Seawater Transfer. J. Fish Biol. 85 (4), 1279–1296. 10.1111/jfb.12473 25159100

[B14] Fang S. Pan J. Zhou C. Tian H. He J. Shen W. (2019). Circular RNAs Serve as Novel Biomarkers and Therapeutic Targets in Cancers. Cgt 19 (2), 125–133. 10.2174/1566523218666181109142756 30411680

[B15] Fu L. Niu B. Zhu Z. Wu S. Li W. (2012). CD-HIT: Accelerated for Clustering the Next-Generation Sequencing Data. Bioinformatics 28 (23), 3150–3152. 10.1093/bioinformatics/bts565 23060610 PMC3516142

[B17] Gong Z. Tian Y. (2010). “Chinese Web Text Classification System Model Based on Naive Bayes,” in International Conference on E-product E-service & E-entertainment, Henan, China, 7-9 Nov. 2010.

[B18] Gumus F. Sakar C. O. Erdem Z. Kursun O. (2014). “Online Naive Bayes Classification for Network Intrusion Detection,” in IEEE/ACM International Conference on Advances in Social Networks Analysis & Mining, Beijing, China, 17-20 Aug. 2014.

[B25] Guohua H Jincheng L (2018). Feature Extractions for Computationally Predicting Protein Post-Translational Modifications. Curr. Bioinformatics 12 (4), 387–395. 10.2174/1574893612666170707094916

[B19] He S. Guo F. Zou Q. Ding H. (2020). MRMD2.0: A Python Tool for Machine Learning with Feature Ranking and Reduction. Curr. Bioinformatics 15 (10), 1213–1221.

[B20] He X. Wang S. Wang R. Dan Z. (2010). “Research of P2P Traffic Identification Based on Naive Bayes and Decision Tables Combination Algorithm,” in Seventh International Conference on Fuzzy Systems & Knowledge Discovery, Yantai, China, 10-12 Aug. 2010.

[B21] Hu Y. Qiu S. Cheng L. (2021). Integration of Multiple-Omics Data to Analyze the Population-specific Differences for Coronary Artery Disease. Comput. Math. Methods Med. 2021, 7036592. 10.1155/2021/7036592 34447459 PMC8384508

[B22] Hu Y. Sun J. Y. Zhang Y. Zhang H. Gao S. Wang T. (2021). Variant Associates with Alzheimer's Disease and Regulates TMEM106B Expression in Human Brain Tissues. BMC Med. 19 (1), 11. 10.1186/s12916-020-01883-5 33461566 PMC7814705

[B23] Hu Y. Zhang H. Liu B. Gao S. Wang T. Han Z. (2020). rs34331204 Regulates TSPAN13 Expression and Contributes to Alzheimer's Disease with Sex Differences. Brain 143 (11), e95. 10.1093/brain/awaa302 33175954 PMC7719023

[B24] Hua T. Zhao Y. W. Ping Z. Zhang C. M. Rong C. Huang P. (2018). HBPred: a Tool to Identify Growth Hormone-Binding Proteins. Int. J. Biol. 14 (8), 957–964. 10.7150/ijbs.24174 PMC6036759 29989085

[B27] Jiao S. Zou Q. Guo H. Shi L. (2021). iTTCA-RF: a Random forest Predictor for Tumor T Cell Antigens. J. Transl Med. 19 (1), 449. 10.1186/s12967-021-03084-x 34706730 PMC8554859

[B28] Lei X. Shanshan J. Jin W. Quan Z. (2021). An In Silico Approach to Identification, Categorization and Prediction of Nucleic Acid Binding Proteins. Brief. Bioinform. 22 (3), bbaa171. 10.1093/bib/bbaa171 32793956

[B29] Lin H. (2020). Development and Application of Artificial Intelligence Methods in Biological and Medical Data. Cbio 15 (6), 515–516. 10.2174/157489361506200610112345

[B30] Liu B. Fang L. Wang S. Wang X. Li H. Chou K. C. (2015). Identification of microRNA Precursor with the Degenerate K-Tuple or Kmer Strategy. J. Theor. Biol. 385, 153–159. 10.1016/j.jtbi.2015.08.025 26362104

[B31] Liu B. Liu F. Wang X. Chen J. Fang L. Chou K. C. (2015). Pse-in-One: a Web Server for Generating Various Modes of Pseudo Components of DNA, RNA, and Protein Sequences. Nucleic Acids Res. W1, W65–W71. 10.1093/nar/gkv458 PMC4489303 25958395

[B32] Liu B. Long R. Chou K. C. (2016). iDHS-EL: Identifying DNase I Hypersensitive Sites by Fusing Three Different Modes of Pseudo Nucleotide Composition into an Ensemble Learning Framework. Bioinformatics 32 (16), 2411–2418. 10.1093/bioinformatics/btw186 27153623

[B33] Liu B. Wang X. Lin L. Dong Q. Wang X. (2008). A Discriminative Method for Protein Remote Homology Detection and Fold Recognition Combining Top-N-Grams and Latent Semantic Analysis. BMC Bioinformatics 9, 510. 10.1186/1471-2105-9-510 19046430 PMC2613933

[B34] Liu B. Liu F. Wang X. Chen J. Fang L. Chou K.-C. (2015). Pse-in-One: a Web Server for Generating Various Modes of Pseudo Components of DNA, RNA, and Protein Sequences. Nucleic Acids Res. 43, W65–W71. 10.1093/nar/gkv458 25958395 PMC4489303

[B35] Liu B. Xu J. Zou Q. Xu R. Wang X. Chen Q. (2014). Using Distances between Top-N-Gram and Residue Pairs for Protein Remote Homology Detection. Bmc Bioinformatics 15 (S2), S3. 10.1186/1471-2105-15-s2-s3 PMC4015815 24564580

[B16] Li W Godzik A(2006). Cd-hit A Fast Program for Clustering and Comparing Large Sets of Protein or Nucleotide Sequences. Bioinformatics 22 (13), 1658. 10.1093/bioinformatics/btl158 16731699

[B36] Liu Z.-P. (2020). Predicting lncRNA-Protein Interactions by Machine Learning Methods: A Review. Curr. Bioinformatics 15 (8), 831–840.

[B37] Liu B (2017). BioSeq-Analysis: a Platform for DNA, RNA and Protein Sequence Analysis Based on Machine Learning Approaches. Brief. Bioinform. 20 (4), 4. 10.1093/bib/bbx165 29272359

[B38] Manavalan B. Basith S. Shin T. H. Lee D. Y. Wei L. Lee G. (2019). 4mCpred-EL: An Ensemble Learning Framework for Identification of DNA N4-Methylcytosine Sites in the Mouse Genome. Cells 8 (11), 1332. 10.3390/cells8111332 PMC6912380 31661923

[B39] Mortezaeefar M. Fotovat R. Shekari F. Sasani S. (2019). Comprehensive Understanding of the Interaction Among Stress Hormones Signalling Pathways by Gene Co-expression Network. Cbio 14 (7), 602–613. 10.2174/1574893614666190226160742

[B40] Niu M. Wu J. Zou Q. Liu Z. Xu L. (2021). rBPDL: Predicting RNA-Binding Proteins Using Deep Learning. IEEE J. Biomed. Health Inform. (99), 1. 10.1109/jbhi.2021.3069259 33780344

[B41] Niu M. Zou Q. Zou Q. (2021). SgRNA-RF: Identification of SgRNA On-Target Activity with Imbalanced Datasets. Ieee/acm Trans. Comput. Biol. Bioinf. 105 (16), 1. 10.1109/tcbb.2021.3079116 33979289

[B42] Polat K. Güneş S. (2009). A New Feature Selection Method on Classification of Medical Datasets: Kernel F-Score Feature Selection. Expert Syst. Appl. 36 (7), 10367–10373. 10.1016/j.eswa.2009.01.041

[B43] Qu K. Han K. Wu S. Wang G. Wei L. (2017). Identification of DNA-Binding Proteins Using Mixed Feature Representation Methods. Molecules 22 (10), 1602. 10.3390/molecules22101602 PMC6151557 28937647

[B44] Qu K. Zou Q. Shi H. (2021). Prediction of Diabetic Protein Markers Based on an Ensemble Method. Front. Bioscience-Landmark 26 (7), 207–221. 10.52586/4935 34340268

[B45] Quan Z. Zeng J. Cao L. Ji R. (2016). A Novel Features Ranking Metric with Application to Scalable Visual and Bioinformatics Data Classification. Neurocomputing 173, 346–354. 10.1016/j.neucom.2014.12.123

[B46] Riaz F. Li D. (2019). Non-coding RNA Associated Competitive Endogenous RNA Regulatory Network: Novel Therapeutic Approach in Liver Fibrosis. Cgt 19 (5), 305–317. 10.2174/1566523219666191107113046 31696817

[B47] Schneider M. (2012). The Annotation of Plant Proteins in UniProtKB. California: Plant & Animal Genome.

[B48] Shen Z. Zou Q. (2020). Basic Polar and Hydrophobic Properties Are the Main Characteristics that Affect the Binding of Transcription Factors to Methylation Sites. Bioinformatics 36 (15), 4263–4268. 10.1093/bioinformatics/btaa492 32399547

[B49] Snow R. W. Guerra C. A. Noor A. M. Myint H. Y. Hay S. I. (2005). The Global Distribution of Clinical Episodes of Plasmodium Falciparum Malaria - Supplementary Information. Nature 434, 214–217. 10.1038/nature03342 15759000 PMC3128492

[B50] Sohm F. Manfroid I. Pezet A. Rentier-Delrue F. Rand-Weaver M. Kelly P. A. (1998). Identification and Modulation of a Growth Hormone-Binding Protein in Rainbow trout (*Oncorhynchus mykiss*) Plasma during Seawater Adaptation. Gen. Comp. Endocrinol. 111 (2), 216–224. 10.1006/gcen.1998.7106 9679093

[B51] Su R. Liu X. Wei L. Zou Q. (2019). Deep-Resp-Forest: A Deep forest Model to Predict Anti-cancer Drug Response. Methods 166, 91–102. 10.1016/j.ymeth.2019.02.009 30772464

[B52] Tan J.-X. Li S. H. Li S.-H. Zhang Z.-M. Chen C.-X. Chen W. (2019). Identification of Hormone Binding Proteins Based on Machine Learning Methods. Math. biosciences Eng. MBE 16 (4), 2466–2480. 10.3934/mbe.2019123 31137222

[B53] Tang H. Zhao Y.-W. Zou P. Zhang C.-M. Chen R. Huang P. (2018). HBPred: a Tool to Identify Growth Hormone-Binding Proteins. Int. J. Biol. Sci. 14 (8), 957–964. 10.7150/ijbs.24174 29989085 PMC6036759

[B54] Wang J. Shi Y. Wang X. Chang H. (2020). A Drug Target Interaction Prediction Based on LINE-RF Learning. Cbio 15 (7), 750–757. 10.2174/1574893615666191227092453

[B55] Wang J. Wang H. Wang X. Chang H. (2020). Predicting Drug-Target Interactions via FM-DNN Learning. Cbio 15 (1), 68–76. 10.2174/1574893614666190227160538

[B56] Wang K. Li S. Wang Q. Hou C. (2018). Identification of Hormone-Binding Proteins Using a Novel Ensemble Classifier. Computing 101 (6), 693–703. 10.1007/s00607-018-0682-x

[B57] Wang X.-F. Gao P. Liu Y.-F. Li H.-F. Lu F. (2020). Predicting Thermophilic Proteins by Machine Learning. Cbio 15 (5), 493–502. 10.2174/1574893615666200207094357

[B58] Wei L. Su R. Luan S. Liao Z. Manavalan B. Zou Q. (2019). Iterative Feature Representations Improve N4-Methylcytosine Site Prediction. Bioinformatics 35 (23), 4930–4937. 10.1093/bioinformatics/btz408 31099381

[B59] Wei L. Su R. Wang B. Li X. Zou Q. (2018). Integration of Deep Feature Representations and Handcrafted Features to Improve the Prediction of N 6 -methyladenosine Sites. Neurocomputing 324, S0925231218306325. 10.1016/j.neucom.2018.04.082

[B60] Xiao Y. Zhang J. Deng L. (2017). Prediction of lncRNA-Protein Interactions Using HeteSim Scores Based on Heterogeneous Networks. Sci. Rep. 7 (1), 3664. 10.1038/s41598-017-03986-1 28623317 PMC5473862

[B61] Yan XY Zhang SW Zhang SY. 2016. Prediction of Drug-Target Interaction by Label Propagation with Mutual Interaction Information Derived from Heterogeneous Network. Mol. Biosyst. 12, 520–531. 10.1039/c5mb00615e 26675534

[B62] Yang H. Luo Y. Ren X. Wu M. He X. Peng B. (2021). Risk Prediction of Diabetes: Big Data Mining with Fusion of Multifarious Physical Examination Indicators. Inf. Fusion 75, 140–149. 10.1016/j.inffus.2021.02.015

[B63] Zeng X. Yuan S. Huang X. Zou Q. (2015). Identification of Cytokine via an Improved Genetic Algorithm. Front. Comp. Sci. 9 (004), 643–651. 10.1007/s11704-014-4089-3

[B64] Zeng X. Zhong Y. Lin W. Zou Q. (2020). Predicting Disease-Associated Circular RNAs Using Deep Forests Combined with Positive-Unlabeled Learning Methods. Brief. Bioinform. 21 (4), 1425–1436. 10.1093/bib/bbz080 31612203

[B65] Zhang J. Ju Y. Lu H. Xuan P. Zou Q. (2016). Accurate Identification of Cancerlectins through Hybrid Machine Learning Technology. Int. J. Genomics 2016 (7-13), 1–11. 10.1155/2016/7604641 PMC4961832 27478823

[B66] Zhang W. Qu Q. Zhang Y. Wang W. (2017). The Linear Neighborhood Propagation Method for Predicting Long Non-coding RNA–Protein Interactions. Neurocomputing 273 (jan.17), 526–534. 10.1016/j.neucom.2017.07.065

[B67] Zhang X. Shi S. Shen J. Zhao M. He Q. (2019). Functional Immunoregulation by Heme Oxygenase 1 in Juvenile Autoimmune Diseases. Cgt 19 (2), 110–116. 10.2174/1566523219666190710092935 31288720

[B68] Zhang Y. Marchant T. (1999). Identification of Serum GH-Binding Proteins in the Goldfish (Carassius auratus) and Comparison with Mammalian GH-Binding Proteins. J. Endocrinol. 161 (2), 255–262. 10.1677/joe.0.1610255 10320823

[B69] Zou Q. Wan S. Ju Y. Tang J. Zeng X. (2016). Pretata: Predicting TATA Binding Proteins with Novel Features and Dimensionality Reduction Strategy. BMC Syst. Biol. 10 (4), 114. 10.1186/s12918-016-0353-5 28155714 PMC5259984

[B70] Zou Q. Lin G. Jiang X. Liu X. Zeng X. (2020). Sequence Clustering in Bioinformatics: an Empirical Study. Brief. Bioinform. 21 (1), 1–10. 10.1093/bib/bby090 30239587

